# Pandemic-Era WIC Participation in Wilmington, Delaware: Participants’ Experiences and Challenges

**DOI:** 10.3390/nu15030520

**Published:** 2023-01-19

**Authors:** McKenna M. Halverson, Allison Karpyn

**Affiliations:** Center for Research in Education and Social Policy, Department of Human Development and Family Sciences, University of Delaware, Newark, DE 19713, USA

**Keywords:** WIC, COVID-19 pandemic, infant formula shortage, inflation, benefit redemption, food insecurity

## Abstract

Special Supplemental Nutrition Program for Women, Infants, and Children (WIC) participants faced unprecedented challenges during the coronavirus disease 2019 (COVID-19) pandemic including financial concerns, a national infant formula shortage, and rising food costs. To mitigate these challenges, the United States Department of Agriculture implemented WIC program waivers and flexibilities aiming to simplify program operations (e.g., remote appointments and food package substitutions). However, little is known about WIC participants’ perceptions of these changes and their impact on in-store benefit redemption. As such, this study aimed to characterize how pandemic-related events impacted Delaware WIC participants’ shopping experiences and program perceptions. The authors conducted semi-structured interviews with 51 WIC participants in Wilmington, Delaware. Survey measures included demographic questions, the Hunger Vital Sign, and open-ended questions regarding WIC program participation experiences during the pandemic. Data were analyzed using a hybrid inductive and deductive coding approach. The results demonstrate that WIC participants benefitted from the pandemic program’s flexibilities. However, they continued to experience burdensome shopping trips as well as concerns about their ability to feed their families due to infant formula shortages and inflation. These findings indicate the importance of extending existing WIC flexibilities and providing continued support for both participants and WIC-authorized retailors.

## 1. Introduction

The coronavirus disease 2019 (COVID-19) pandemic resulted in considerable economic and psychological hardship for many families [1,2,3,4,5]. In response, a series of actions were taken by the United States (U.S.) government and its agencies to strengthen the safety net [6]. The Special Supplemental Nutrition Program for Women, Infants, and Children (WIC), which is the third largest federal nutrition assistance program in the U.S. serving approximately 6.2 million individuals each month, was one such program that received additional funds and flexibilities during the COVID-19 pandemic [7,8]. WIC provides low income, nutritionally at-risk pregnant and postpartum women and infants and children up to the age of five with access to a monthly supplemental food package as well as nutrition education, healthcare referrals, and breastfeeding support [7]. The WIC food packages contain a variety of healthy food items, with specific brand and package size requirements, tailored to participants’ needs (e.g., 16 oz package of 100% whole wheat bread) [9].

A large body of previous research demonstrates that WIC participants often face difficulties with in-store benefit redemption [10,11,12,13,14,15,16,17,18,19,20]. Several factors have been shown to contribute to these redemption challenges including a lack of accurate, consistent shelf labeling [10,11,17,18,19,20,21], low stock of WIC-approved foods [11,15,16,20,22,23], issues at the checkout (i.e., perceived stigma and long waiting times) [10,11,17,24], confusion about WIC-eligible items (i.e., brands and sizes) [17,24], and inconsistencies between the register and the information participants receive from the program/WIC apps, particularly following program and policy changes [20,22,24]. These challenges were exacerbated by the COVID-19 pandemic and related issues such as global grocery retail shortages and rising food and household costs [1,25,26]. Specifically, the retail industry struggled to keep pace with shopper demand for staple products during the pandemic, and global shortages of home goods and food were well documented [25,26]. Such limitations were reported to be particularly challenging for families with low incomes, such as WIC participants, given that staple products were frequently purchased first, leaving fewer options for those with less money to spend or those attempting to identify WIC-approved brands and package sizes [21,27].

Additionally, in 2022, an infant formula shortage occurred which worsened existing retail stressors and impacted WIC families across the U.S. [28,29,30,31]. Two major factors contributed to this shortage: global supply chain issues stemming from the pandemic and the temporary shutdown of an Abbott plant, one of the main formula manufacturers in the U.S., due to formula safety issues [30]. Estimates from 2018 demonstrate that participants consumed a significant portion (~56%) of infant formula in the U.S. [32]. WIC’s crucial role in the infant formula market rendered this population particularly vulnerable to the consequences of the shortage.

To mitigate the challenges associated with WIC participation during the COVID-19 pandemic, the U.S. Department of Agriculture (USDA) made several modifications to program operations [8]. For example, they implemented program service delivery flexibilities to include remote benefit issuance and physical presence waivers, allowing potential and active participants to complete the required certification visits through online appointments [8,33]. Additionally, they made food package adjustments to permit brand and size substitutions to enable participants’ access to a broader range of potential product selections. To expand access to infant formula for WIC families during the crisis, the USDA also approved non-contract formulas and implemented flexibilities to container size requirements [34].

Although many of these pandemic-related challenges are well documented in the popular press, few studies have empirically evaluated the ways in which these events, as well as the WIC waivers and flexibilities, impacted participant’ experiences during the COVID-19 pandemic. Therefore, this qualitative study aimed to examine the perceptions and challenges of WIC participants during this time to enhance our understanding of the conditions which support or detract from healthy food access for this vulnerable population.

## 2. Materials and Methods

### 2.1. Participants and Procedure

Between March and June of 2022, we recruited 53 WIC-eligible individuals from Wilmington, Delaware, via study flyer distribution at a large regional supermarket chain, community-based partner organizations, childcare centers, and churches [35]. The eligibility criteria for the study were as follows: Delaware resident, at least 18 years of age, English-speaking, contribute to household food purchasing, and participated in the WIC program between March 2020 and June 2022. We dropped two participants from our analyses following interviews during which it was revealed that they did not meet the eligibility criteria. Therefore, our total sample consisted of 51 participants.

The participants completed semi-structured phone interviews lasting approximately 20− to 30 min. The semi-structured interview guide, which inquired about WIC participants’ experiences during the pandemic, was created in partnership with the Village Tree, Incorporated (Inc.), and Conscious Connections, Inc., two of the research teams’ community partners in Wilmington, Delaware (See Table 1 for representative interview questions). As compensation for their participation in the survey, the participants received a USD 20 ShopRite gift card. The Institutional Review Board at the University of Delaware reviewed and approved all of the study procedures.

### 2.2. Measures

#### 2.2.1. Demographics

The following questions were administered to assess the participants’ demographic information: gender, race, ethnicity, educational level, income level, employment status, relationship status, parent age, number of children currently participating in the WIC program, pregnancy status, number of people living in the household, participants’ WIC participation status, SNAP participation over the last year, and current SNAP participation.

#### 2.2.2. Hunger Vital Sign

The Hunger Vital Sign [36] is a two-item food insecurity screener with the following statements/questions: (1) “Within the past 12 months we worried whether our food would run out before we got money to buy more”, and (2) “Within the past 12 months the food we bought just did not last and we did not have money to get more”. The participants could respond to questions with “often true”, “sometimes true”, or “never true”. Responses of “often true” and “sometimes true” for either or both questions were coded as food insecure whereas responses of “never true” were coded as food secure. The Hunger Vital Sign has been shown to be a comparable tool to the 18-item US Household Food Security Scale and it has high sensitivity (97%), specificity (83%), and convergent validity [36].

#### 2.2.3. WIC Experiences during the COVID-19 Pandemic

A series of open-ended questions evaluating WIC participants’ experiences with appointments and benefit redemption during the COVID-19 pandemic were included as part of the semi-structured interview guide (Table 1).

#### 2.2.4. Data Analysis

We used Statistical Package for Social Sciences (SPSS) version 27 to analyze the participants’ demographic characteristics and Dedoose (9.0.46 OSX) to analyze qualitative data. All of the interviews were audio-recorded and transcribed using Rev.com. To analyze the qualitative data, we employed a hybrid inductive and deductive thematic analysis approach [37]. We employed deductive coding strategies to develop a codebook based on semi-structured interview guides to understand WIC participants’ experiences and challenges during the COVID-19 pandemic (e.g., in-store redemption strengths and challenges and infant formula shortages) and inductive coding strategies to identify themes that emerged from the data. The primary investigator (MMH) engaged in preliminary coding efforts and discrepancies were resolved by the study coauthor (AK).

#### 2.2.5. Researcher Positionality

Both the study principal investigator (MMH) and coauthor (AK) are White, middle-class women who have not participated in the WIC program. The authors recognize that their race and socioeconomic status have afforded them opportunities and privileges commonly denied to families with low incomes or historically marginalized racial and ethnic groups. The authors are dedicated to ensuring their research findings reflect the voices of individuals from these communities and inform policies and practices rooted in equity.

## 3. Results

The participants’ demographic characteristics are listed in Table 2. All 51 participants were female (100%) and were predominately Black (70.6%), had incomes under $30,000 (68.6%), were employed full- or part-time (56.8%), were food insecure (76.5%), and had an average of 2.3 children (SD = 0.89) participating in the WIC program.

WIC participants reported five major themes regarding their experiences with the program during the COVID-19 pandemic. These themes included: (1) perceptions of remote appointments, (2) WIC product availability and identification, (3) WIC app utilization and challenges, (4) infant formula shortage concerns, and (5) altered shopping practices to account for inflation. Each theme and two exemplifying quotes for each category are provided in Table 3 below.

### 3.1. Theme 1: Perceptions of Remote Appointments

The first theme emerging from the data demonstrated that WIC participants highly valued the shift from in-person to remote appointments and services during the COVID-19 pandemic. Specifically, the participants reported that remote appointments were easier to manage and that they benefitted from not having to leave the house and rush to in-person appointments. For example, Respondent 20 described,

*“The appointments, I feel like got a lot better. Before the whole pandemic situation, we would have to sit in the office, which I completely understand. There’s always a wait and everything, but I feel like the phone call is more convenient versus having to rush to get to the appointment. […] It makes it a lot easier on my behalf”.*

Similarly, Respondent 19 said, *“Everything was done over the phone which was actually good because instead of me having to pull all of my children out, I got to do what I needed to do over the phone, and I really liked that”.*

However, a few participants stated that although they liked the remote appointments, they missed certain aspects of the in-person appointments. For example, Respondent 37 expressed,

*“I would say appointments got easier [because] now they are over the phone. I don’t have to be personally in there. I guess the bad part is that my child doesn’t get to really see the doctor and get to know them”.*

Additionally, a few participants reported communication challenges with the remote appointment format. For example, Respondent 44 said,

*“Well, my phone that was pretty good, but I do know that I had an appointment and I waited for the call. Nobody called me. And when I called back, they were like, well, she should be giving your call shortly. […] So that was an issue for me”.*

Despite these challenges, participants generally perceived the switch to remote appointments positively.

### 3.2. Theme 2: WIC Product Availability and Identification

A second key theme relates to participants experiences with WIC product availability and identification in stores during the pandemic. Participants approved of the additional food and package size substitution options available to them and said that they enhanced their ability to identify WIC-eligible items in stores. As Respondent 49 said,

*“They did make things a little bit easier […] because of the food shortage and stuff like that. […] Also, a lot of things that we weren’t able to purchase before, we [were] able to purchase through the pandemic for a certain brand of baby food and stuff like that. So, I thought that was pretty cool cause sometimes you would go there and the brand that you were looking for wouldn’t be there, but they all had other brands right next to it. So, it made things a little bit easier when they approved certain things”.*

Respondent 49 also expressed, *“I see WIC trying to like add a lot of options, add some new things […] that we can purchase. I just appreciate [being] in the program because it helps when it’s needed”.*

However, despite these flexibilities, the participants noted that a few key issues, including inconsistent WIC product shelf labeling and a low stock of WIC-approved items, made identification of WIC-eligible items in stores difficult. Regarding a lack of consistent shelf labeling, Respondent 10 described,

*“Yes. It would be very beneficial if all, and I mean, all of the stores that participated in the WIC program for the state had the WIC-identifying things on their aisles. A lot of the stores still miss having the WIC identifier. They went away from having the very, very small “W” to just having the big, big, bold “W”, but some stores still fall short of actually putting those up on items and having enough stuff in the items per time”.*

Similarly, Respondent 52 reflected,

*“Sometimes it would be better if the stores themselves would clearly have more like of the black and white WIC signs. You know how they normally have like little WIC signs that they put on their items? Those are helpful because sometimes it can get confusing. Like WIC-approved items or whatever, or if I don’t have WIFI, like if I didn’t have my phone on me to look on the app to scan it or something. Then you get it to register and it’s not the right kind, or it’s not approved. […] If the stores themselves were to mark the items better, it would be easier”.*

The participants also stated that there was often a low stock of WIC-eligible foods in stores, and many had to shop at multiple stores to fully redeem their benefits. For instance, Respondent 26 recalled, “*When it came to the store, I wasn’t able to get certain things that I was normally able to get. Certain milk and yogurts*”.

Respondent 10 also commented, *“Honestly, most of them didn’t even allow you to get anything and if they didn’t have it, they just didn’t have it. And then you just had to go to another store”.*

Product availability also varied both between and within stores over time. For example, Respondent 17 said,

*“It depends on what store you go to. Cause [if] you go to [Supermarket 2], the yogurt that comes on your WIC, you have a choice between three different flavors, but then you go to other stores that are closer to me like [Supermarket 3] or [Supermarket 1], or even the [Supermarket 4], [Supermarket 4] you could barely do anything there”.*—Respondent 17

Likewise, Respondent 19 reported, “*Certain stores approve [different] items than other stores and it’s constantly changing. So, it’s kind of a hassle for me. Sometimes I’ll go to the store to get one thing and then the following week it’s not approved, so I have to get something else*”.

Confusion about WIC-eligible brands and size was also common among participants. For instance, Respondent 3 remarked,

*“The bread selection is confusing to me. I did not know the longer loaves are not the loaves of bread that are acceptable. It is the smaller loaves, I guess, wheat. It gets confusing in a lot of the stores like at the register they tell me that’s not actually the WIC one I’m supposed to grab. It happens at [Supermarket 1] all the time”.*

In addition to the main benefit redemption-related challenges, a couple of participants expressed that they wished their unused WIC benefits would roll over each month. For example, Respondent 31 voiced,

*“The biggest thing that I do not like about WIC […] was that it doesn’t roll over to the next month. I had four kids in three years, so I was getting a lot of WIC. I had like tons and tons of milk that just disappeared after the month was over, which I could have used into the next month and stuff. I just don’t like how it doesn’t roll over”.*

### 3.3. Theme 3: WIC App Utilization and Challenges

The third key theme emerging from the data reflects the participants’ experiences using the WIC app. Most participants reported that they enjoyed using the WIC app and that it simplified the benefit redemption process by helping them to better understand their benefits, particularly when items weren’t properly labelled on the shelves. For example, Respondent 40 said,

*“I just got the WIC app, and it shows me the update of my benefits and so I really like that. So, I always know what’s in there and how much I can spend and stuff and what’s covered. That app is very helpful. In the past I would just look at these for the stickers and sometimes I don’t know if they got knocked down or fell off or what, but sometimes it wouldn’t be posted on certain aisles for certain items. But now because of the app, I know specifically what I can and cannot get”.*

Respondent 52 also stated, “*I love the new app. I have the app on my phone, and I like how you can scan the barcodes in the store to see […] if it’s a WIC-approved item or not*”.

However, at the checkout, a few participants experienced frustration in that the items on their WIC app were not consistent with the information they received at the vendors’ registers. For instance, Respondent 35 reported,

*“I have the WIC app. I go to scan a certain product that I want, and it says WIC-approved. [But] it tells me at the time that it’s not a selection I can do. So, I’m not sure if it’s something in the system that’s not activated because I did do a concerned comment to the nutritionist, and she said sometimes the person that is in charge of […] WIC doesn’t activate it in their computer system to have it approved by the card. So that is one of the issues”.*

### 3.4. Theme 4: Infant Formula Shortage Concerns

The fourth key theme derived from the data reflects participants’ concerns about the infant formula shortage. Mothers shared that they experienced considerable challenges locating WIC-approved formula and that the lack of available formula was a major stressor for themselves and their families. The participants worried about their ability to feed their children. For example, Respondent 46 expressed,

*“I cried and had panic attacks. People think I’m crazy because my baby is five months, and he needs to eat. I can give him food, but he still needs formula. WIC don’t really tell you where to go to get it. I guess no one really knows. […] It’s still a little frustrating cause I’m still looking for it”.*

Many participants also voiced frustration about retailer limits on the number of formula cans families could buy. Issues such as a lack of transportation and time inhibited them from making frequent shopping trips. As Respondent 51 said, *“I know the store [Supermarket 1] people [usually] get nine cans but the lady was trying to give me three because of the shortage. We don’t drive now so this is inconvenient—giving three cans per person instead of nine”.*

Additionally, several participants stated that even with the formula limits, they had difficulty finding infant formula and they had to go to multiple stores to identify WIC-approved brands. Respondent 50 said, “*So the stores that we’re allowed to get stuff at, they be out of stock. The stores that we can’t use the WIC, they had it in stock”.*

The Similac recall eroded one participants’ trust in WIC-approved formula. Specifically, Respondent 14 said, “*I still didn’t feel comfortable giving [Similac] to my newborn. It was recalled. […] So, when that happened, I haven’t even been using [WIC for my kids]. I’ve just been buying like the ready to feed off the EBT*”.

Despite these concerns about formula stocking and identification during the pandemic, Respondent 31 stated that she benefitted from the flexibilities in formula choice provided by the WIC program during the shortage.

*“With the recall on Similac that just happened not too long ago, it is good that I can go in and swipe my WIC card for an alternative choice, similar to the milk that is on my WIC. I don’t think I was ever able to do that”.*

However, although some participants benefitted from these WIC formula flexibilities, others were not aware of their substitution options and faced confusion about which formulas were approved. For example, Respondent 44 expressed,

*“Nobody was helping me with WIC on what other brand(s) would I getting without the Similac? So, I was buying the other brand out of pocket. So, I haven’t used my WIC in almost two months for formula. So yeah, it has impacted my groceries”.*

Additionally, Respondent 39 said *“Maybe y’all should like open it all up for more for different brands and not just Similac”* indicating that they were also not aware of the WIC formula flexibilities during the pandemic.

### 3.5. Theme 5: Altered Shopping Practices to Account for Inflation

The fifth key theme that emerged from the data describes the ways in which the participants shifted their shopping practices in response to higher food and household costs resulting from inflation. Many participants mentioned concerns about these rising costs including Respondent 49 who remarked, *“I think the prices are ridiculous, […] so it’s really upsetting”* as well as Respondent 38 who stated, *“It’s horrible. You need to, even with getting food stamps, like that stuff is still high”.*

Many participants expressed that they were forced to alter their shopping habits due to rising prices. Respondent 35 said,

*“Depending if they’re on sale, I try to get ‘em on sale, and sometimes they’re not on sale, and the kids are asking, ‘Mommy, can I have this?’ and I’m like ‘Well, let’s see the ad to see if it’s on sale and then we can get more of what you like. If it’s not, we only get a little bit and try to the make the amount last throughout the month until I get my reload.’ So, I try to budget”.*

Respondent 11 said her family had to reduce her family’s meat purchases due to inflation, “*I’ve also, we’ve kind of minimized how much meats we actually eat because the price of that is also like kind of crazy for the amount of pounds that you get*”.

Additionally, inflation negatively impacted Respondent 17’s families’ healthy eating habits,

*“One orange is USD 1.15. And [I’m] like well, we can’t do this [fruit and vegetable] this week, so we’ll have to try something different because of the amount that’s available. […] I got three growing boys. My 11-year-old is overweight, he’s going on all these diets cause there’s not enough fruits and vegetables available to them because of the prices. So, what are they gonna do? They’re gonna turn to junk”.*

Additionally, several participants said they had to seek out food from other sources such as food banks or schools to make ends meet. One participant even changed jobs to find food. Specifically, Respondent 25 stated,

*“I really had to reduce myself to working at a place that sells food, so I get 50% off. So, I could just eat at work and not have to worry about buying groceries. Like it was that bad. I started a business, so hopefully by the time I don’t get any more benefits […] everything should be okay. The WIC is definitely a great help. […] I’m not too proud, help is help”.*

Therefore, inflation had major impacts on the ways in which WIC participants shopped for and obtained food, as well as the quality of the food their families consumed during the pandemic.

## 4. Discussion

This study evaluated Wilmington, Delaware, WIC participants’ experiences navigating the program during the COVID-19 pandemic. Our findings demonstrate that participants benefitted from the pandemic-related program modifications implemented by the USDA, including remote appointments and food package flexibilities (i.e., brand and package size expansions). Specifically, participants perceived the remote appointments to be more efficient, and safer, than in-person appointments. Research conducted prior to the pandemic indicated that certain aspects of in-person appointments (e.g., long wait times, leaving the house with children) were burdensome for participants and made appointment attendance challenging [12,17]. More recent research, which was conducted after pandemic-related WIC program modifications were enacted, found that many participants were satisfied with remote options and prefer them over traditional in-person appointments [12,13,23]. Our results support these findings.

Although most participants in our sample had positive perceptions of the remote appointments, a few participants recalled that they faced difficulties communicating with WIC staff over the phone and missed certain aspects of in-person appointments (i.e., forming relationships with doctors). Mixed perceptions of remote appointments have also been identified in prior studies [12,33]. Thus, research suggests that moving forward, implementation of hybrid WIC service and appointment options may be warranted [23,38].

Regarding in-store benefit redemption during the pandemic, participants expressed gratitude for the flexibilities to WIC-approved brands and package sizes. They also valued the WIC app, stating that it simplified their shopping experience. These positive perceptions of the WIC app are important as research shows that WIC app usage is associated with higher benefit redemption rates [39].

Despite the implementation of program modifications during this time, benefit redemption challenges were common. Consistent with the results of previous research, many participants had difficulty finding WIC-approved items in stores due to a lack of shelf labeling and low stock of WIC foods [10,11,15,16,17,18,19,20,22,23,24]. Some participants also experienced confusion when determining which brands and sizes were WIC-eligible [17,24]. Additionally, some participants communicated that inconsistencies between their WIC app and items approved by vendors’ registers at the checkout were a major source of frustration when attempting to redeem their benefits. A desire for unused WIC benefits to roll over each month was also mentioned.

In addition to these in-store benefit redemption challenges, the participants experienced an infant formula shortage in 2022, which had serious implications on their ability to feed their infants. Many participants said they had to shop at multiple stores to find WIC-approved formula and that transportation limitations and time constraints inhibited their ability to find formula. Some participants even expressed fears about having more children in the future due to their potential inability to feed them. Although flexibilities enabling the purchasing of non-contract formula were enacted during this period [34], several participants indicated that they were unaware of these substitution options, indicating a need for improved communication strategies surrounding WIC policy and practice changes [11,22].

Furthermore, rising food and household bills due to inflation have also been shown to pose a substantial challenge for WIC participants by impacting their ability to feed their families [11,23]. This finding held true in the present sample. To account for these increasing prices, the participants altered their shopping practices by searching for bargains and buying fewer of certain items (i.e., meats, fruits, and vegetables). Additionally, the participants reported obtaining food from other sources (e.g., food banks and schools) to account for inflation, which aligns with previous research [23].

This study has some important limitations to address. First, our sample was relatively homogenous with respect to race (70.6% Black) and geography (Wilmington, Delaware), so our findings may not reflect the experiences of other WIC participant populations, with less generalizability than multi-state samples; future studies with more diverse samples are warranted. Quantitative studies examining participants’ altered shopping strategies, benefit redemption rates, and retail location preferences due to the infant formula shortage and inflation are also needed to understand the impact of financial stressors and food shortages on WIC families.

## 5. Conclusions

The results of this qualitative study provide important insight into the experiences of WIC participants in Wilmington, Delaware, during the COVID-19 pandemic. Our findings demonstrate that WIC participants benefitted from the pandemic program’s flexibilities and modifications including remote benefit issuance, physical presence waivers, and food package substitution options. However, the parallel supply chain issues, which resulted in a lack of available products, including formula, and rises in food costs due to inflation contributed to challenging benefit redemption experiences for the participants. Our results provide insight into factors that may help or hinder this population’s future benefit redemption experiences. Additionally, these findings indicate the importance of permanently extending existing WIC flexibilities and the need to provide tailored support to participants and WIC-authorized retailors during public health crises.

## Figures and Tables

**Table 1:** Representative interview questions.

(1) Can you tell me a little bit about your WIC benefits during the COVID-19 pandemic? (Probe: Did you experience any changes to your appointments or shopping experiences during the pandemic? Did you encounter any challenges with the WIC program during the pandemic?)
(2) Has inflation impacted your families’ grocery shopping experiences? If so, how? (3) Have you been unable to get the formula you need due to a shortage? If so, what was your experience like? (4) Is there anything else you would like to share about your experience with the WIC program or any recommendations you would like to make to improve the program moving forward?

**Table 2:** Sample characteristics (*N* = 51).

	Mean (SD)
Parent Age	30.88 (7.01)
Number of People in Household	4.39 (2.01)
Number of Children Currently Participating in the WIC Program	2.3 (0.89)
	%
Gender (Female)	100
Race	
Black	70.6
White	11.8
Other	2.0
Ethnicity (Hispanic/Latino)	21.7
Educational Level	
Less than a High School Degree	9.8
High School Degree	49.0
Some College	31.4
Bachelor’s Degree	7.8
Doctorate/Professional Degree	2.0
Income Level	
Under $30,000	68.6
$30,000–$60,000	21.6
$60,001–$90,000	3.9
Over $90,000	2.0
Prefer Not to Say	3.9
Employment Status	
Employed Full-Time	39.2
Employed Part-Time	17.6
Unemployed and Looking for Work	17.6
Unemployed and Not Looking for Work	9.8
Unable to Work/On Disability	9.8
Other	5.9
Relationship Status	
Single	66.7
Live at Home with Partner or Spouse	29.4
Other	3.9
Food Insecure	76.5
Mother Currently Participates in the WIC Program (*N* = 44) *	29.5
Mother is Currently Pregnant	19.6
Currently Enrolled in SNAP (*N* = 42) *	56.9
Enrolled in SNAP Over the Last Year (*N* = 43) *	70.6
Struggled to Find Infant Formula During the Shortage (*N* = 8 *)	50.0
Impacted by Inflation (*N* = 44) *	84.1

*Note.* * Question added during survey administration.

**Table 3:** Qualitative themes and exemplar quotes.

**Theme #1: Perceptions of Remote Appointments**	
Remote appointment and service benefits	“[Appointments] did get honestly just easier instead of having to always come in they were able to just call me and confirm all the information they needed to and then my benefits were transferred. So, it actually helped to not get a delay”.—Respondent 11 “I think it was pretty helpful not [having] to go out during the pandemic because he [the baby] was still so new. I had just had him, so it was convenient to be able to do everything over the phone and it was helpful”.—Respondent 1
**Theme #2: WIC Product Availability and Identification**	
Approval of COVID-19-related food package changes	“During the pandemic I was pregnant, so I got the pregnancy WIC. […] A lot of changes at that time that they were offering. Different foods that they didn’t offer before. So that was good”.—Respondent 35 “I liked the increase in fresh fruit and vegetables. That was very helpful. […] We liked the extra additions”.—Respondent 53
Lack of accurate in-store labeling	“They don’t really have the stuff labeled, like they used to, so it’s a headache”.—Respondent 18 “A lot of the grocery stores, they need to get up on their labeling cause a lot of times people don’t know what’s WIC until you get to the front. They [say] oh, that [didn’t] go through. I have that problem all the time”.—Respondent 51
Low stock of WIC items and shopping at multiple stores to fully redeem benefits	“When I’m doing my WIC for the week, I have to go to three different stores”.—Respondent 19 “It was hard sometimes actually finding WIC things on the shelves. Like, some stores actually were giving you other things in place of the WIC items if they didn’t have it, but that was only certain stores”.—Respondent 10
**Theme #3: WIC App Utilization and Challenges**	
Positive perceptions of the WIC app	“Well, I like that I can access it on my phone. So, I used the app. That makes it easier to know what I have left […] instead of keeping the receipts around because that’s too much after a while. […] For example, I didn’t know the milk switched until after the fact. I’m thinking I still was getting 2%, but then I had to switch it to 1%. So that helps to like, be able to scan that to prove if the store doesn’t have it labeled”.—Respondent 7 “It’s just so much easier now that it’s on an app instead of like trying to use the coupons and stuff”.—Respondent 2
Inconsistencies between the WIC app and vendors’ registers	“I don’t want to go to register and have to keep guessing. Even if I scan it, sometimes he’ll tell me that it’s not the right thing. So, like, sometimes I just end up not being able to use my benefits […]. So, it’s a little problem category I run into”.—Respondent 21 “I don’t really know the stuff that we’re allowed to get. […] I don’t feel like it’s clear enough on the app. And a lot of times the app doesn’t work. Like when I scan stuff, it’ll say that it’s WIC-approved, but then when I go up to the register, they’re telling me that it’s not approved”.—Respondent 18
**Theme #4: Infant Formula Shortage Concerns**	
Retailor restrictions and issues finding formula in stores	“Yeah [the Similac recall] that’s been a problem. You go to the store to pick up the WIC [formula] and they only allow you to get only four and some stores don’t even have it at all. And then sometimes I have to buy it with my food stamp card because of that issue”.—Respondent 22 “There [were] times that I was going to like six to seven different stores a day. […] And even with WIC, if they did have it in stock, [my son] was only allowed to get four cans. Which even if you got the four cans, the WIC they wouldn’t even let you buy more, you know, without using the WIC so I was just constantly going to the grocery store every two days”.—Respondent 44
Concerns about the ability to feed future children	“It actually makes me scared about having another baby. […] I had a problem with my son in breastfeeding”.—Respondent 39 “I’m glad that I don’t have to deal with Similac [any] longer, but it’s just a little skeptical and concerning to make sure that my son isn’t involved in that shortage. Or if I ever do to decide if I want another child, that’s something that’s gonna be concerning”.—Respondent 37
**Theme #5: Altered Shopping Practices to Account for Inflation**	
Acknowledgement of increasing grocery prices	“Most definitely. Everything is expensive now. So, like blueberries that were like USD 2.20 is now USD5.45. So yeah, it’s crazy. So like WIC definitely helps. Helps a lot”.—Respondent 46
	“I guess because of the cost of living is up and the healthy stuff costs more and the junk food costs less. But the price of everything’s going up. Right now, instead of like USD 0.99 per pound of chicken, it’s all up to like USD 2 to USD 3 per pound of chicken. The cucumbers are almost a USD 1.79 apiece. Instead of USD 0.60 or USD 0.80, they’re almost USD 2 a cucumber now. Everything’s going up and being less available”.—Respondent 16
Searching for bargains	“Yes, I go to more stores now chasing bargains and things of that nature. And I check the circular before I go”.—Respondent 53
	“Yes. So, I’m very particular now about the way in which I shop for food. I try to save as much money as possible. Because I don’t wanna break the bank”.—Respondent 40
Obtaining food from other sources	“Definitely. Well now I had to, we have to sign up with the Delaware Food Bank. We get the box delivered stuff. They have helped tremendously, but those are canned and the sodium. […] Everything [is] expensive right now”.—Respondent 52 “I’m glad like these schools have these things now that like hand these bags that have two little things of milk, two juices, two cereals, some grapes and like a little snack or something. Or they have these, at least the elementary schools, they have snack time that they bring him the fruits and vegetables from schools because families can’t afford it. And that’s why I told anyone if you can, if you are, if you qualify, go for it”. —Respondent 17
Impact of increasing bills on grocery shopping experiences	“Yes, certain bills [gas and rent] have changed and gotten pricier. With the extra food money that we are receiving either with the EBT or the WIC it is all a lot of extra help, it is great help we need. With gas and bills our money that we receive from working is going straight to bills so if we didn’t have that extra money, it would be very hard to pay for all eight of us”.—Respondent 20 “The prices are getting higher and higher. Even the gas can you put USD 25 gas and you don’t even get half a tank, so it’s hard to get from A to B. So, there’s no gas and you try to ask the extra neighbor, can you, you know, take me there and they want money and everything’s about money and it’s hard. It’s really, really hard. So, when my oldest daughter was going to do the grocery shopping, we just go with her. We all do it together”.—Respondent 22

## Data Availability

The data presented in this study are available upon request from the corresponding author.
